# Global health concern on the rising dengue and chikungunya cases in the American regions: Countermeasures and preparedness

**DOI:** 10.1002/hsr2.1831

**Published:** 2024-01-24

**Authors:** Ranjan K. Mohapatra, Priyadarshini Bhattacharjee, Dhruv N. Desai, Venkataramana Kandi, Ashish K. Sarangi, Snehasish Mishra, Ranjit Sah, Amani Ahmed AL. Ibrahim, Ali A. Rabaan, Kudrat E. Zahan

**Affiliations:** ^1^ Department of Chemistry Government College of Engineering Keonjhar Odisha India; ^2^ Department of Clinical Medicine Cambridge University Hospitals NHS Foundation Trust Cambridge UK; ^3^ Department of Pathobiology University of Pennsylvania Philadelphia Pennsylvania USA; ^4^ Department of Microbiology Prathima Institute of Medical Sciences Karimnagar Telangana India; ^5^ Department of Chemistry Centurion University of Technology and Management Balangir Odisha India; ^6^ School of Biotechnology KIIT Deemed University Bhubaneswar Odisha India; ^7^ Department of Microbiology Tribhuvan University Teaching Hospital, Institute of Medicine Kathmandu Nepal; ^8^ Department of Clinical Microbiology Dr. D. Y. Patil Medical College, Hospital and Research Centre, Dr. D. Y. Patil Vidyapeeth Pune Maharashtra India; ^9^ Deparment of Pharmacy Jubail General Hospital Jubail Saudi Arabia; ^10^ Molecular Diagnostic Laboratory Johns Hopkins Aramco Healthcare Dhahran Saudi Arabia; ^11^ Department of Medicine, College of Medicine Alfaisal University Riyadh Saudi Arabia; ^12^ Department of Public Health and Nutrition The University of Haripur Haripur Pakistan; ^13^ Department of Chemistry Rajshahi University Rajshahi Bangladesh

**Keywords:** chikungunya, dengue, epidemiology, healthcare, seasonal infection, vector‐borne disease

## Abstract

**Background and Aim:**

Severe morbidity and mortality due to seasonal infectious diseases are common global public health issues. Vector‐borne viral illnesses like dengue and chikungunya overload the healthcare systems leading to critical financial burden to manage them. There is no effective drug or vaccine currently available to control these two diseases.

**Methods:**

The review was formulated by incorporating relevant reports on chikungunya and dengue in the Americas regions through a comprehensive search of literature that were available on dedicated scientific publication portals such as PubMed, ScienceDirect, and Web of Science.

**Results:**

The strategies of public health administrations to control largely the mosquito vectors during tropical monsoon seem to be effective. Yet, it seems practically impossible to completely eliminate them. The mosquito vector disseminates the virus via transovarian route thereby internalising the virus through generations, a reason behind reappearing and recurring outbreaks. The numerous factors associated with industrialisation, urbanisation, population density, and easy transboundary movements appear to have contributed to the spread of vectors from an endemic region to elsewhere.

**Conclusion:**

The article made a state‐of‐affair comprehensive analysis of the rising dengue and chikungunya cases in the tropics, particularly the tropical Americas, as a human health concern, the countermeasures undertaken and the overall preparedness. The viral transmission is a hard situation to tackle as the vector survives in diverse temperature and ecology, is resistant to insecticides, and the unavailability of drugs. Better vector‐control measures and improved understanding of the reemerging arboviral infections could offer an extended reaction time to counter outbreaks, and minimise associated morbidity/mortality.

## 1 INTRODUCTION

The geographically expanding arboviral diseases like dengue and chikungunya is a major global public health concern.^1^ Dengue alone accounts for the largest number of infectious disease cases in the Americas, manifesting itself as an epidemic every 3–5 years.^2^ Although these two infectious diseases are endemic to most of the Central America, South America and the Caribbean, its increased transmission and spread are noticed beyond its previously reported transmission regions. Dengue transmission might intensify to most part of the southern hemisphere soon due to the building up of conducive weather conditions for the mosquitoes to thrive and proliferate, experts opine. The Americas reported 2.8 million dengue cases in 2022 as compared to 1.2 million cases in 2021, an above twofold increase.^2^ Chikungunya cases reported so far also exhibit a similar trend. There are unprecedented rise in the incidents of chikungunya in Paraguay and dengue in Bolivia.^2^, ^3^, ^4^ High incidents of chikungunya‐associated meningoencephalitis add to the healthcare concerns.

Nearly three million suspected and confirmed (2,997,097) dengue cases in the American regions with 1302 deaths (at 0.04% case fatality rate) are reported by the WHO as by July 1, 2023. Brazil, Peru and Bolivia outnumbered the dengue cases till date in 2023. The Americas regions reported a total of 3,123,752 combined suspected and confirmed arboviral disease cases in 2022.^2^ Of this, 90% (2,809,818) cases were dengue and 9% (273,685) were of chikungunya. A total of 2,809,818 dengue case was a twofold increase in confirmed cases and 1290 deaths an almost threefold increase in 2022 as compared to 1,269,004 confirmed cases and 437 deaths in 2021.^2^ Bolivia reported dengue virus type 2 (DENV 2) as the predominant serotype, while Paraguay reported DENV 1 and DENV 2, and Peru reported DENV 1, DENV 2, and DENV 3 serotypes. With 223,782 cases in 2020, Paraguay reported its first‐ever large dengue outbreak. The highest cumulative dengue case reported from Peru was 68,290, in 2017.

The American regions reported a total of 113,447 chikungunya cases with 51 deaths as of March 4, 2023, a fourfold increase in the number of confirmed cases and deaths as compared with the previous year data of 21,887 cases and 08 deaths.^2^ Brazil reported a total of 50,103 chikungunya cases during the period, an 83% increase in cases compared to 2022 (Table 1).^2^ Bolivia reported a total of 593 chikungunya cases which is an astounding 11‐fold increase compared to 2022. Peru has reported 97 chikungunya cases in 2023 so far, a nearly threefold increase compared to the previous year.

**Table 1:** The reported dengue and chikungunya cases in the American regions.^a^

Country	Dengue		Chikungunya	
	Confirmed case	Death	Confirmed case	Death
Bolivia	1st Jan–24th June ‘23		1st Jan–11th Mar ‘23	
	133,779	77	593	‐
Paraguay	1st Jan–4th Mar ‘23		2nd Oct ‘22–4th Mar ‘23	
	686	‐	40,984	46
Peru	1st Jan–1st July ‘23		1st Jan ‘23–4th Mar ‘23	
	188,326	325	97	‐
Argentina	1st Jan–1st July ‘23		1st Jan ‘23–12th Mar ‘23	
	126,431	65	341	‐
Brazil	1st Jan–1st July ‘23		1st Jan–11th Mar ‘23	
	2,376,522	769	50,103	6 confirmed; 23 suspected
Colombia	1st Jan–24th June ‘23		‐	
	50,818	29	‐	‐
Nicaragua	1st Jan–24th June ‘23		‐	
	56,780	01	‐	‐

^a^ *Source*: WHO; https://www.who.int/emergencies/disease-outbreak-news/item/2023-DON448; https://www.who.int/emergencies/disease-outbreak-news/item/2023-DON475.

Due to the continuous rise of dengue and chikungunya cases in the American regions, there is a pressing need to address the health concern for the sake of global health and wellbeing. The article aimed at carrying out a detailed analysis of the compiled and synthesised data from various online sources, as detailed above, and drew conclusions and recommendations to counter the potentially endemic arboviral transmissions of multiple diseases that are on the rise in especially the American regions.

## 2 MATERIALS AND METHODS

To conduct this narrative review, literature search from Scopus, Pubmed, Web of Science, and online data/information of the various health agencies of global relevance like WHO and CDC was conducted. One hundred and seventy relevant literature/data were found, out of which 41 were shortlisted considering the focus of the article. The rest were discarded for the lack of focus or relevance from the article perspective. The data/information from the selected/shortlisted articles were reviewed, compiled, analysed, synthesised and are presented to develop an article with seamless flow, and draw rational and justified comments, and conclusion for the ease of appraisal and understanding by a reader. The data analyses carried out included the reasons behind the unexpected rise of Chikunguyan cases in the American regions and the contributing environmental factors. The citations in the article were then compiled manually and are presented in the “references” section as per the Journal guidelines.

## 3 EPIDEMIOLOGY

Infective spread through mosquito bite is the basis of arboviral illnesses like dengue and chikungunya. Almost four billion people in the tropical and subtropical regions are prone to it, posing a health hazard (https://www.who.int/news-room/events/detail/2022/03/31/default-calendar/global-arbovirus-initiative).

### 3.1 Dengue

Dengue is a disease primarily affecting the urban and peri‐urban settings, especially in the tropical and subtropical continents.^5^ *Aedes aegypti* mosquitoes and *Aedes albopictus* (to a lesser degree) are the major carriers. The transmission cycle of dengue virus in the vectors is complex as evident by its transfer from nonhuman primates to humans, and the human‐to‐human spread during outbreaks. About 75% of dengue infections remain asymptomatic and 5% of the infected population might have severe manifestation of the disease. Delays in diagnosis and initial treatment are primarily the cause of death among 20% of the infected.^6^

The virus (DENV) responsible for causing dengue is a RNA virus of Flaviviridae family.^5^ It is an enveloped virus consisting of a positive sense RNA. Dengue virus has three structural proteins—capsid (C), membrane (M), and envelope (E), and several nonstructural (NS) proteins like NS1, NS2A, NS2B, NS3, NS4A, NS4B, and NS5. The data of confirmed viral dengue are available since the 18th century (1780). Complex pathophysiology of the viral infection in humans as evident from the antibody‐dependant enhancement (ADE), and a continual alteration of genetic material are the reasons for the nonavailability of disease‐control prophylactics such as vaccines.^7^

The pathogen has more than three different yet similar variants (DENV‐1, DENV‐2, DENV‐3, and DENV‐4).^5^ Successive episodes of dengue viral infection with different serotypes leave patients at higher risks of developing severe dengue that could include septic shock or respiratory difficulty owing to hypovolemic shock, internal bleeding, organ damage and fatality. An exposure to one serological variant conferred long‐term immunity to the homologous serotype but not to the other serotypes.

Although currently there are no particular therapy for dengue, prompt incident discovery, recognition of a significant disease alarm mechanism, and effective crisis intervention remain essential action‐plan to avoid severe illness, mortality, and fatalities reduction to about a percent.^5^ The “Region of the Americas” is compiling statistical information on dengue since 1980. This pathogen has nonetheless propagated over the majority of the territory. Having far greater than three million incidental reports, involving twenty‐eight thousand extreme situations and over seventeen hundred fatalities, in the year 2019, infection rates reached a record high.

As of now, dengue is endemic in over 100 countries, of which the Americas, Western Pacific and South‐East Asia regions are the most seriously affected and represent a high disease burden. Denguvaxia [chimeric Yellow fever virus‐Dengue virus (CYD‐TDV)], DENVax/TAK‐003, TV005, V180, and D1ME100 DNA vaccines are few vaccine candidates against dengue at various clinical trial stages.^8^, ^9^, ^10^, ^11^ Of these, Denguvaxia is the first USFDA‐approved vaccine for emergency use.

### 3.2 Chikungunya

Chikungunya, another virus spread by mosquito, induces high temperature and excruciating soreness. It was initially identified in 1952 in southern Tanzania, in the form of an epidemic. Female mosquito variants of *A. aegypti* and *A. albopictus*, that may spread dengue and Zika, seem to be the significant prevalent vectors of Chikungunya virus (CHIKV). While there could be bursts of occurrence in the wee hours of the day and later after midday, these insects attack and transmit all throughout.

The incubation period of CHIKV is 5–7 days, similar to DENV. The similar clinical presentation of CHIKV and DENV as also other endemic diseases like malaria, measles, leptospirosis, and so forth makes it difficult to differentially diagnose them without resorting to high‐end molecular techniques such as reverse‐transcription polymerase chain reaction (RT‐PCR) for specific laboratory confirmation.^12^

Depending on how long the pathological changes last, chikungunya is abrupt, intermittent or persistent. Although it is uncommon for a clinically critical case to escalate to fatality, people at the extremities of their lifespan seem to be more likely to fall serious. The illness is characterised by an abrupt appearance of symptoms, commonly followed by painful and incapacitating joint problems or arthralgia lasting for an appreciable time period. Neurological side‐effects like Guillain‐Barré syndrome and meningoencephalitis have since been confirmed. Majority of the patients recover fully with lifetime protection.

Chikungunya in the newborn has been clearly noticed. CHIKV pathogen usually is not teratogenic and is not reported to be transmitted from the mother to the foetus. However, the transmission risk is expectedly the maximum if a woman is contaminated during the preterm phase, where the longitudinal rate of infection is as high as 49%.^13^ Babies often have no symptoms at conception until high temperature, shakeup, rashes and peripheral oedema manifest. Additionally, women contracting the infection at peripartum phase may experience haemorrhagic manifestations, heart issues and neurological illness (like meningoencephalitis, white matter abnormalities, cerebral oedema and intracranial haemorrhage). Deviations in the liver function, lower platelet and lymphocyte counts, and low prothrombin levels are among others as diagnosed in the laboratory. Neurodevelopmental illness in the newborn frequently leads to prolonged disability. There seems to be no proof that such virus may spread through breastfeeding. Reported first in December 2013 and a catastrophic spread in 2014, the genesis of chikungunya is traced to the US. The disease has extended geographically ever since.

## 4 PUBLIC HEALTH MEASURES

The World Health Organisation (WHO), in coordination with individual territorial healthcare systems, is assisting epidemic disaster preparedness in the United States.^2^ The assistance includes surveillance, laboratory and clinical management, advocacy and planning. Promoting medical and monitoring capabilities is important to execute an Integrated Management Strategy (IMS‐Arbovirus) to prevent and control arboviral illness. Efficient integrated vector surveillance and control through regular release of recommendations and providing monitoring resources also seem important. Several AI and non‐AI computer‐assisted assessments, prevalence newscast, bolstering etiological oversight of transmissible and communicable diseases like zika, dengue, and chikungunya are suggested. WHO promotes scientific capability expansion to facilitate real‐time incident identification and characterisation. In close association with Bolivia, Colombia, Chile, Ecuador, Peru, and Venezuela, it also works together to improve the local government‐controlled medical capabilities to treat and control arboviral illnesses through Global Arbovirus Initiative (GAI) that was introduced in 2022. GAI is an embedded master decision focusing on the vulnerability assessment, pandemic avoidance, readiness, recognition, rebuttal, and bipartisan cooperation to counter growing and reemerging arboviral public health crisis and projecting the looming pandemic. Encouraging waste mitigation action‐plan by local bodies and using personal protection at households could be an effective addition.

Proliferating mosquito vectors have developed resistance to pyrethroid compounds applied to control them. *A. albopictus* mosquito, a potential carrier of dengue, chikungunya, and zika viruses, developed deltamethrin resistance.^14^ As mosquito vectors are equally prevalent in the tropic, subtropic and temperate regions, it is essential to enhance the understanding on their survival and spread and devise strategies to control and possibly eliminate them.^15^ Seven epidemics that occurred in Brazil were assessed, and it was found that the CHIKV showed spatial heterogeneity, wherein the increased viral spread in a particular geographical region led to herd immunity and reduced infection rates.^16^ However, the trend could not be associated with recurrences of outbreaks, attributable to the reemergence of viral infections after a long gap and thus the loss of neutralising antibodies as one of the factors. Lack of an animal model to carry out studies was a major cause for the relatively low interest in CHIKV research, leading to the unavailability of drugs and vaccines against it.^17^, ^18^, ^19^

## 5 INCREASING THE PUBLIC KNOWLEDGE, AWARENESS AND PRACTICE

Devising strategies to control and prevent DENV and CHIKV are particularly essential to ensure global public health and wellbeing. Being arthropod‐borne and mosquito‐transmitted, both viruses are seasonal viral infections. DENV is attributed to increased morbidity and mortality particularly in resource‐limited countries. Control and prevention strategies include eliminating the mosquitoes, protective measures against mosquito bite, and vaccination.^5^, ^20^ An earlier study in Delhi, India reported that although 80% had the awareness of transmission of DENV, a meagre less than 10% was aware of potential symptoms and simple preventive measures like restricting mosquito breeding.^21^ Another study observed that intervention programmes could increase awareness, knowledge and perception about DENV transmission, symptoms, vector control and prevention measures.^22^ Recent study also revealed that an increase in knowledge regarding DENV and other vector‐borne infections among the public will contribute to reduced infection rates.^23^

## 6 VECTOR CONTROL

DENV outbreak reports are frequent especially in urban setup, most being due to improper sanitation, like water logging and shabby urban conditions.^24^ High population density contributes to easy viral transmission. Ensuring cleanliness and controlling mosquito breeding by local municipalities is essential.^5^, ^24^ Apart from cleanliness and sanitation, mosquito control has been attempted by measures like using mosquito repellent chemicals like diethyltoluamide to control mosquito proliferation, and biological control like infecting the mosquitoes with *Wolbachia* bacterium, using gene editing like CRISPR (clustered regularly interspaced short palindromic repeat) to alter the sex of the female mosquito, and employing fish like *Gambusia* and copepods that feed on mosquito larvae.^25^, ^26^, ^27^, ^28^

## 7 VACCINATION

Vaccination against dengue and chikungunya appears to be most promising control measure. There are only few universally approved vaccines against DENV and CHIKV currently. DENV is a greater public health threat compared to CHIKV. DENV is in multiple serotypes, and two vaccines (Dengvaxia and Qdenga) are approved for emergency use among travellers. CHIKV vaccine is easy to develop due to its limited serotypes, but the disease is plagued with uncertain distribution due to its sporadicity combined with unpredictable emergence and reemergence. Some CHIKV vaccines are currently under phase III trial.^29^ However, if a vaccine becomes available in future, vaccination may not be enough to eliminate the virus. Thus, it is essential to integrate different aspects including vaccination, vector control and exploratory research to tackle these infections.^30^, ^31^

## 8 SURVEILLANCE STRATEGIES

DENV control and elimination must include surveillance strategies like extensive well‐directed research including the impact of environment or climate on DENV transmission and bite‐proof school dresses to protect children from mosquito bite, sensing and monitoring systems and infection prediction models.^32^ Surveillance of mosquitoes using gravid oviposition sticky (GOS) traps and dengue nonstructural 1 (NS1) antigen test could potentially predict the infections.^33^ Regardless of conventional surveillance in population, sero‐surveillance to detect antibodies against the infecting microbe for DENV could increase the understanding about the region‐wise disease burden.^34^ Applying digital surveillance technologies like integrated digital environmental surveillance system (SILIRA) for surveys on epidemiology, vector‐based entomology and interview to assess disease burden and devise DENV control strategies was advocated.^35^ Such integrated surveillance of infected cases and screening for vectors that harbour DENV shall help control and prevent the diseases.

### 8.1 RISK ASSESSMENT

Owing to the vast distribution of *Aedes* spp. (mostly *A. aegypti*), pathogens causing these discussed diseases have spread in the Americas region for generations. These arboviruses can spread locally, where the carriers exist, and susceptible populace and could reach places through contaminated travellers (imported cases).^2^, ^5^ Being arboviruses, population in locations with the insect vectors are at risk. Most severe effects could be observed where the arboviral infection programmes lack sufficient funding to carryout the action‐plan. Despite the fact that dengue and chikungunya are endemic throughout the majority of the tropical and subtropical regions in the Americas and the Caribbean, a rise in chikungunya infections elsewhere is noticed that is worrisome. Dengue propagation has been reportedly active in 2023.^2^, ^36^

The challenging disease concerns and potential risks resulting in insufficient disease management may be owed to, delayed diagnosis due to nonspecific symptoms of dengue and chikungunya that resemble to illnesses like zika and measles, overburdened medical facilities in areas dealing with high backlog and other co‐occurring diseases, and the consequences of COVID‐19 pandemic on the IT/telecommunication infrastructure. Implications of increased incidents in the region shall need to consider numerous variables. These include nation's capacity for a synchronised preventive approach and patient care, initiation of arbovirus cases in the southern peninsula, rising mosquito intensities due to interrupted vector surveillance and control during the COVID‐19 pandemic, and demography size that is sensitive to arbovirus infections particularly in regions where the pathogens tend to spread fast.^2^, ^5^, ^20^

Intriguing fact is the potential greater incidence of acute meningoencephalitis linked to chikungunya in Paraguay. The reason that is increasing the prevalence of neurologic illness that has an unusual clinical appearance is currently unknown; initially discovered in Brazil in 2014, it is endemically growing. The East‐Central‐South‐African (ECSA) lineage has been decoded. The propagation of chikungunya virus among the serologically uninformed populace may be a reason for spread in future. Widespread *Aedes* spp. distribution in the Americas region can possibly lead to trans‐border transmission of dengue and chikungunya. The adjacent countries such as those close to Paraguay and Bolivia, particularly the highly connected ones, may experience expedited dengue illnesses and heightened chikungunya incidence. Further, the hot and humid summer in the Southern part could impact vector behaviour and could facilitate arbovirus transmission.^2^, ^36^

These elevated factors are at regional scale owing to extensive involvement of the associated mosquito vector species (especially *A. aegypti*), the ongoing risk of serious significant morbidity and mortality, and the augmentation outside the chronic endemic regions, wherein the general public particularly the risk groups and healthcare professionals don't seem to be cognisant and are recombinant illiterate. The lack of necessary items to control and prevent, a scarcity of supplies for laboratory assays and testing, and the requirement to retrain the field teams and healthcare professionals are other issues as mentioned by the member States.^2^, ^36^ Due to favourable meteorological conditions for vector spawning during the first semester of the year, high propagation rates are anticipated in the region in the coming months.

## 9 RECOMMENDATIONS

Incessant rainfall and poor sanitation facilitate the reproduction of arbovirus vectors. The socioeconomy like inadequate habitats could be favourable for vector to replicate.^37^ The Amazon tropical rainforest which is house to one of the long rivers that cover a huge area of South America could be an ideal environment for the vector. About 60% of Amazon is in Brazil, while the rest is shared among Bolivia, Colombia, Peru, Ecuador, Venezuela, Guyana, Suriname, and French Guiana. It could be an ideal habitat for the vector especially owing to its large adjoining marshy land area, both in length and breadth. From Table 1, it is observed that most of the dengue cases are observed from such countries. Intensive and focussed research on this to assess the hypothesis is warranted. Research to assess vertical transmission in mosquitoes and whether these vectors adapt to fresh environments as also the impact of global warming and climate change on the vector to establish there are also needed.^38^, ^39^

Monitoring and management measures for *Aedes* spp. need a lot of attention to protect the population in the region from the most competent vector.^2^, ^5^ Transmission rate could be reduced through targeted effective tracking and management approaches.^5^ Reducing the transmission and mortality requires early diagnosis of disease severity and appropriate medical care. Community participated appropriate protective measures may be initiated in companies, classrooms and residential areas, like insect repellents, insecticide‐treated bed nets during biting hours (daytime and early evening hours) and dressing with long sleeves and pants to prevent mosquito bites.^5^ No specific dengue and chikungunya antiviral therapy is available and supportive treatment like hydration and antipyretics are resorted to as clinical therapy measures.

As symptoms of these arboviruses may be quite similar, it may be difficult to clinically define them. As dengue and zika viruses cross‐react serologically, ill‐defined serological prediction may lead to epidemiological oversight resulting in inadequate case management. Thus, RT‐PCR molecular diagnostics is advisable. Member States in the Americas region must remain remarkably alert and prepared to step up efforts for prevention, early detection, diagnosis and control of arboviruses.^2^ It includes educating and coaching health professionals on how to recognise instances and associated problems, identifying severe‐illness risk groups, managing critical cases to prevent death, and actively coordinating and sharing knowledge across borders. With abundant occurrence of arboviral diseases in the previous 3 years, an upswing in arbovirus cases particularly in the southern peninsular region in the first half of 2023 is anticipated. Such an increase may be followed by a high transmission among the vulnerable communities due to heat‐waves this season as experienced in the northern South America, Central America and the Caribbean.^37^

New tools and techniques for case surveillance, vector monitoring and improved laboratory testing techniques for quick response in arbovirus surveillance to avoid fresh outbreaks or to minimise the number of critical cases are necessary.^40^ For effective surveillance, it is required to adopt a multidisciplinary approach and integrate them. A seamless and effective risk mitigation communication by timely informing the public about these arboviral diseases and encourage them to adhere to prevention and control measures are essential to ensure a healthy wellbeing.

## 10 CONCLUSION

Dengue and chikungunya outbreaks by the vector‐borne arboviruses appear inevitable particularly in the endemic geographical regions. Due to several reasons including the ability of vectors to survive in an array of temperature and environmental conditions, it is difficult to get rid of the viral transmission. The resistance of the viruses to insecticides appears to hamper the conventional control measures. Unavailability of drugs against the diseases further cripples the healthcare infrastructure to manage the patients. Discovery of specific drugs against these viruses is a plausible solution, and research and development of vaccines against them in future that work successfully and sustainably is another. Better vector‐control measures and improved understanding of reemergence of vector‐borne arboviral infections could extend the response time for preparedness to counter a future outbreak, while minimising the associated morbidity and mortality.^41^

## AUTHOR CONTRIBUTIONS

**Ranjan K. Mohapatra**: Conceptualization; supervision; writing – original draft. **Priyadarshini Bhattacharjee**: Writing – original draft. **Dhruv N. Desai**: Data curation; validation. **Venkataramana Kandi**: Validation; writing – original draft. **Ashish Sarangi**: Investigation. **Snehasish Mishra**: Writing – review & editing. **Ranjit sah**: Writing – review & editing. **Amani Ahmed AL. Ibrahim**: Writing – review & editing. **Ali Rabaan**: Writing – review & editing. **Kudrat Zahan**: Writing – review & editing.

## ETHICS STATEMENT

The authors have nothing to report.

## TRANSPARENCY STATEMENT

The lead author Ranjan K. Mohapatra, Kudrat‐E‐Zahan affirms that this manuscript is an honest, accurate, and transparent account of the study being reported; that no important aspects of the study have been omitted; and that any discrepancies from the study as planned (and, if relevant, registered) have been explained.

## Data Availability

The authors have nothing to report.
